# The grazing activity of *Acrobeloides* sp. drives phytate mineralisation within its trophic relationship with bacteria

**DOI:** 10.21307/jofnem-2021-021

**Published:** 2021-02-25

**Authors:** Mercedes García-Sánchez, Mathilde Souche, Carlos Trives-Segura, Claude Plassard

**Affiliations:** Eco&Sols, INRAE, Université de Montpellier, CIRAD, Institut Agro, IRD, Montpellier, France

**Keywords:** *Acrobeloides* sp, Bacterial proliferation, Nematode migration, Micro-food web interactions, Phytase-producing bacteria, Phosphorus cycling

## Abstract

The microbial loop has been suggested as an alternative route for better utilization of phytate, a poorly available P source to plants. We hypothesized that bacterial grazer activity might dramatically enhance bacterial migration and proliferation, increasing the probability of phytate hydrolysis by bacterial phytases and, thus, phytate mineralization and release of free phosphate. We tested this hypothesis in a two-compartment system with a solid medium containing phytate or free phosphate as the source of P. Two bacterial species, *B. subtilis* 168 or *Bradyrhizobium* sp., with or without bacterial grazing nematodes belonging to *Acrobeloides* sp. previously fed on each of the bacterial species, were inoculated at a single point in the medium. Whatever the P source, nematode migration within both zones allowed the proliferation of bacteria. However, *B. subtilis* 168 was more efficient in using phytate than *Bradyrhizobium* sp. since the highest bacterial cell density and free phosphate concentrations were reached by *Acrobeloides* sp. fed on *B. subtilis* 168. The grazer activity seemed to be crucial to enhance phytate mineralization, despite *Acrobeloides* sp. showing a higher preference to feed on *Bradyrhizobium* sp. This study provides new insights into the effects of bacterial grazer activity on phytate mineralization.

Phytate (myo-inositol-hexakisphosphate) is often the dominant form of organic phosphorus (Po) found in soils and may constitute up to 50 to 80% of the total Po in soil (Turner, 2007; Turner et al., 2002). To be used by plants and microorganisms, phosphate groups of phytate must be hydrolyzed by phytases, which are released into the external medium or at least into the cell wall space. Hence, phytate mineralization is a prerequisite to convert Po into plant-available forms, which are free orthophosphate ions (Pi). Based on their structural differences and varied catalytic properties, four classes of phytases have been proposed. These include: (i) members of histidine acid phytases (HAP), found in fungi, plants, and bacteria; (ii) ß-Propeller phytase (BPP), found mainly in bacteria; (iii) cysteine phytase (CPhy), found in bacteria from anaerobic environments; and (iv) purple acid phytase (PAP), found in plants (Mullaney, 2007; Mullaney and Ullah, 2003; Singh et al., 2020). Up to now, plants grown in axenic conditions have been shown to exhibit a poor capacity to use phytate as the sole source of P (Hayes et al., 2000; Richardson et al., 2000, 2001a, b), suggesting a low capacity to release phytases into the external medium.

In contrast to plants, many different bacteria carry genes encoding phytases, representing one important group of phytate-mineralizing bacteria (Lim et al., 2007; Neal et al., 2017). This includes the gram-positive, endo-spore-forming bacteria such as *Bacillus* sp., which have been widely studied for their ability to secrete a phytase belonging to the BPP class (Greiner et al., 2007; Jain et al., 2016; Jorquera et al., 2008, 2011; Kumar et al., 2017; Shin et al., 2001) and for their potential use as fertiliser enablers in agriculture (Hill et al., 2007; Richardson and Simpson, 2011; Singh et al., 2020). The BPP phytase specifically hydrolyzes calcium phytate by releasing *myo*-inositol triphosphate and three Pi groups as end products (Jain et al., 2016; Kumar et al., 2017; Mullaney, 2007). Other than bacteria belonging to the *Bacillus* genus, recent studies revealed that the abundance of gram-negative bacteria belonging to the *Bradyrhizobium* genus harboring the *pho-D* gene, putatively involved in soil Po mineralization, increased significantly following organic manure input (Bi et al., 2020). Surprisingly, the *Bradyrhizobium* genus displayed the highest relative abundance among all other bacterial genera analyzed in soil samples collected under non-leguminous plant species such as beech or spruce trees (Nacke et al., 2016). Hence, besides their capacity to be associated with legumes to ensure the biological nitrogen fixation in nodules, *Bradyrhizobium* bacteria could also play a large role in Po mineralization. So far, no phytase activity has been reported for this bacterial genus, though the genome of *B. japonicum* (strain USDA6^T^) contains a gene encoding a protein with an esterase-like activity of the phytase family (Kaneko et al., 2011), thus raising the question of the possible phytase capacity of this bacterium.

Given their abilities to mobilize efficiently recalcitrant P, inoculants containing selected microbes have been widely proposed (Menezes-Blackburn et al., 2018), with some of them commercially available (Owen et al., 2015). However, the sole inoculation of phytate-mineralizing bacteria may not be beneficial to the plant, as shown in the agarose medium (Irshad et al., 2012) or in soil (Ranoarisoa et al., 2020). These results suggest that P mobilization by bacteria is not efficient enough because they immobilize the released Pi into their biomass (Pistocchi et al., 2018; Spohn et al., 2018), making it non-available to plants, as previously observed by Khan and Joergensen (2009).

Soil bacterivores, mostly represented by protozoa and nematodes, are ubiquitous at sites with high soil microbial activity, indicating their important role driving ecosystem function as a key component of soil diversity (Trap et al., 2016). For example, bacterial grazers play a major role by releasing nutrients sequestered in bacterial biomass in the rhizosphere (Bonkowski et al., 2009; Villenave et al., 2004), and/or by influencing the microbial abundance (Fu et al., 2005) and composition. Although the importance of predation by bacterial grazers has long been recognized, the potential effects of predation remain poorly defined. Bacterial grazers have food preferences, grazing more on gram-negative than on gram-positive bacteria (Djigal et al., 2004; Shtonda and Avery, 2006). This leads to an important but unexplored hypothesis that bacterial grazers may affect the total bacterial composition and specific functional groups such as those involved in P cycling in different manners since the interaction between bacteria and their grazers being the first step of *the microbial loop* might determine the rate of mineral nutrient cycling in the rhizosphere (Jiang et al., 2017).

A study conducted in the rhizosphere of *Pinus pinaster* grown with insoluble mineral P as the sole P source revealed that the inoculation of bacteria and their grazers improved plant P uptake compared to seedlings not inoculated or inoculated with bacteria alone (Irshad et al., 2011). To explain their results, the authors proposed that bacterial P could be released into the medium from bacterial grazing activity (Irshad et al., 2011). Therefore, the strategy based on the exploitation of the microbial loop following the bacterial ability to mobilize phytate might be a promising strategy for better plant phytate utilization. In agreement with this hypothesis, Irshad et al. (2012) reported strong positive effects of the bacterial grazers belonging to *Rhabditis* sp. and *Acrobeloides* sp. feeding on *B. subtilis* on plant P utilization from phytate supplied as the sole source of P. These results were mainly attributed to the ability of nematodes to unlock the bacterial P previously taken up as free orthophosphate resulting from phytate hydrolysis by the BPP phytase secreted into the medium by *B. subtilis*. However, compared to the sole inoculation of bacteria, the bacterial grazers could also enhance the phytate mineralization by increasing the migration of bacteria throughout the medium, thereby increasing the phytase release and phytate hydrolysis. Also, the efficiency of the microbial loop could strongly depend on bacterial quality since bacterivores fed preferentially on Gram-negative bacteria than on Gram-positive ones (Blanc et al., 2006; Liu et al., 2017). In this study, we addressed these hypotheses by evaluating the predation activities of *Acrobeloides* sp. on two bacterial strains, *B. subtilis* 168 and *Bradyrhizobium* sp., differing in morphological features, size, and cell wall structure (Gram+ and Gram−). Bacterial strains were inoculated alone or co-inoculated with *Acrobeloides* sp. within a two-compartment system to measure the bacterial growth and the bacterial grazers migration on two P sources that were phytate or free orthophosphate ions. Also, the efficiency of Pi release from phytate was evaluated when the medium was inoculated with bacteria alone or with bacterial-feeding nematodes.

## Material and methods

### Bacterial strains

The strain of *Bacillus subtilis* 168 used in this experiment was obtained from the German Collection of Microorganisms and Cell Cultures (DSMZ 23778), while the *Bradyrhizobium* sp. was kindly provided by the Laboratory of Tropical and Mediterranean Symbiosis (LSTM) located in Montpellier, France. *B. subtilis* 168 and *Bradyrhizobium* sp. strains were plated on Luria-Bertani (LB) and maintained at 4°C.

For the co-inoculation experiment, a bacterial inoculum was prepared by growing *B. subtilis* 168 and *Bradyrhizobium* sp., respectively, in 20 mL of LB or Yeast Extract Manitol (YEM) medium on a shaker (150 rpm) for 48 hr. The medium was centrifuged (8,000*g*, 10 min), and the bacterial pellet was washed three times in NaCl (1%) solution to produce an inoculum free of Pi. The bacterial suspension was prepared to give a cell density of 10 × 10^6^ cells mL^−1^.

### **N**ematode populations

Populations of *Acrobeloides* sp. nematodes (as in Irshad et al., 2012) were isolated from the topsoil collected under natural savanna at the experimental station of Lazaina (18°46′55 59° S, 47°32′46 3°N, 1,274 m altitude, Madagascar). The soil was a Ferrasol, with a sandy-clay texture and a pH of 5.5, and its complete description is given in Ranoarisoa et al. (2018). The nematodes were maintained on a solid medium containing 1% agar, 3 g L^−1^ Tryptic Soy Agar (TSA) supplemented with cholesterol (5 µg L^−1^), and inoculated with *Escherichia coli*. In order to produce monoxenic populations of *Acrobeloides* sp. fed on *B. subtilis* 168 or *Bradyrhizobium* sp., the eggs of a single gravid female were sterilized using the methodology previously described by Irshad et al. (2011) with some modifications. After hatching the eggs in sterile water, the larvae were collected and inoculated in TSA plates where *B. subtilis* 168 or *Bradyrhizobium* sp. were grown for 48 hr. Bacteria and nematodes were incubated for three weeks at 28°C to induce nematode reproduction. After three weeks, or when the plates contained plenty of nematodes, including gravid females and eggs, the inoculum of nematodes was prepared. The nematodes were first removed from the breeding TSA plates by washing the surface with 5 mL of a sterile NaCl solution (1%) subsequently poured in a 15 mL tube and concentrated by centrifugation (2,000 *g*, 5 min). Thereafter, the pellet containing the bacterial grazers, eggs, and bacteria was suspended in 2 mL of the sterile NaCl solution (1%). Active nematodes (without eggs) were selected using the Cobb sieving method (Van Bezooijen, 2006) with modifications because the entire process was carried out in sterile conditions, under a laminar flow cabinet. We used a 30 µm stainless steel mesh sieve covered with a Kleenex^®^ tissue previously autoclaved twice at 48 hr intervals (121°C, 20 min) and placed it in a sterile glass Petri dish containing 20 mL of sterile distilled water. The nematodes were allowed to migrate through the tissue into the water overnight. The water was recovered in a polypropylene tube and supplemented with distilled water until a final volume of 50 mL was reached. The nematode inoculum was prepared to reach a density of 1,000 nematodes mL^−1^.

### **C**o-inoculation experimental design

**Figure 1:**
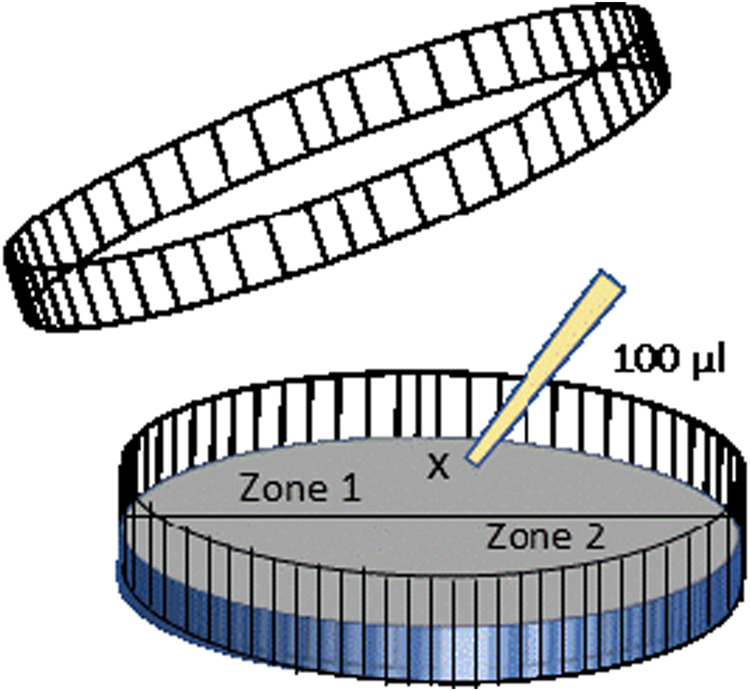
Scheme of the experimental design used to follow the effect of bacterial grazer displacement. Petri dishes (90 mm diameter) were filled with agarose medium containing 1 mM of sodium phytate or inorganic phosphate. The inoculum (100 µL) was placed at a single point of the dish, marked ‘x’. After three weeks, Petri dishes were open and the agarose was separated in two parts called zones 1 and 2.

The effects of nematode migration on bacterial populations and phytate mineralization were quantified in 90 mm Petri dishes in which the solid medium was virtually separated in two identical zones by tracing a line on the lid and called zone 1 and 2 (Fig. 1). Petri dishes were filled with 25 mL of a nutrient medium containing 1.0% agarose (Sigma ref A7431), 55 mM glucose, 2 mM KNO_3_, 2 mM MgSO_4_.7H_2_O, 4 mM CaSO_4_, 50 μgL^−1^ thiamine hydrochloride, 0.5 ml L^−1^ 1% Fe citrate, 50 mM MOPS pH 7, 0.2 ml L^−1^ Morizet and Mingeau (1976) solution of micronutrients, 5 μg L^−1^ cholesterol, and 1 mM phytate (inositol hexakisphosphate sodium salt, Sigma ref P0109) or 1 mM Pi (KH_2_PO_4_) as the sole source of P. The medium without phytate or Pi and cholesterol was autoclaved (115°C, 40 min) and cooled to 55°C before adding a filtered (0.2 μm pore size) sterilized solution of cholesterol (5 mg mL^−1^), phytate (0.1 M, pH adjusted to 7), or Pi (0.1 M). The experiment was launched by placing the inoculum consisting of 100 µL of solution containing nematodes (1,000 individuals mL^−1^) or bacteria (10 × 10^6^ bacterial cells mL^−1^) or 100 µL of sterile water at a single point in the Petri dishes located in the upper part of the defined zone 1 (Fig. 1).

Five treatments were set up: (i) co-inoculated with *Acrobeloides* sp. fed on *B. subtilis* 168, (ii) co-inoculated with *Acrobeloides* sp. fed on *Bradyrhizobium* sp., (iii) inoculated with *B. subtilis* 168, (iv) inoculated with *Bradyrhizobium* sp., and (v) non-inoculated (water addition, control treatment). Five replicates were established for each treatment in the Petri dishes with phytate or Pi as the sole source of P, resulting in a total of 50 plates. The Petri dishes were incubated horizontally at 28°C and were inspected every five days. The experiment was stopped after three weeks because the whole plates were colonized by the nematodes at that time.

### Sampling and analytical procedures

At harvest, bacterial grazers, bacteria, and free Pi were carefully separated from the agarose contained in the two zones of the Petri dishes. The agarose was first cut into two pieces corresponding to zone 1 and 2. Each zone of the agarose was placed in a 50 mL polypropylene tube with 20 mL of distilled water and slightly shaken by hand for 15 min to extract (i) bacteria, (ii) bacterial grazers, and (iii) free Pi into the water solution. Thereafter, the water solution was filtrated to separate bacterial grazers from bacteria and Pi through a nylon sieve with 30 µm pores where the nematodes were retained. The nylon was placed in a 50 mL polypropylene tube and rinsed with 15 mL of distilled water to get all the nematodes into the solution. They were then counted under a stereomicroscope using adequate dilution. Subsequently, the filtrated solution, containing bacterial suspension and free Pi, was centrifuged (8,000*g*, 10 min). After centrifugation, the supernatant containing free Pi was transferred into a 20 mL polypropylene tube and kept at 4°C until Pi quantification using the green malachite method (Ohno and Zibilske, 1991). Finally, the bacterial pellet was carefully re-suspended in 1 mL of distilled water and used to enumerate bacterial cell density with a haemocytometer after adequate dilution.

### Statistical analysis

Unless otherwise stated, the results are given as mean±standard error (*n* = 5). Statistical analysis was carried out using SPSS software version 19.0. Data normality was first tested using the Levene test. As all data were normal, one-way ANOVA was used, followed by Tukey’s HSD post hoc to analyze the differences between means.

## Results

### Spatial expansion of nematodes according to P source and bacterial species

**Figure 2:**
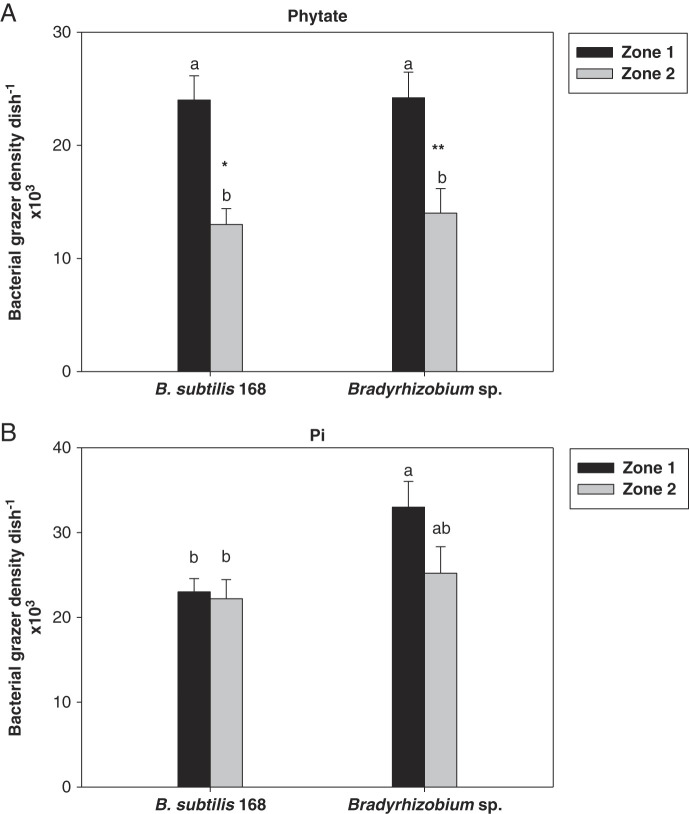
Bacterial grazer populations of *Acrobeloide*s sp. previously fed on *B. subtilis* 168 or *Bradyrhizobium* sp. measured throughout the zones 1 and 2 after inoculation at a single point located at the top of zone 1 and grown either with phytate (A) or Pi (B) as the P source. Data are means (±SE, *n* = 5). The same lowercase letters indicate a lack of significance (*p* < 0.05) within the same zone of the Petri dish. Stars above a bar indicate a significant difference between the two P sources (*p < 0.05; **p < 0.01) for the same zone.

When phytate was supplied as the sole P source, the size of *Acrobeloides* sp. populations did not differ according to the bacterial strain but were roughly twice as large within zone 1 than within zone 2 (Fig. 2A). In contrast, nematode populations fed on bacteria grown with Pi were significantly higher on *Bradyrhizobium* sp. (+40%) than on *B. subtilis* in zone 1. In zone 2, they were not significantly different from those counted in zone 1 for either *B. subtilis* 168 or *Bradyrhizobium* sp. and were the same for the two bacterial strains (Fig. 2B). They were also significantly higher than those counted in zone 2 of the phytate medium (Fig. 2).

Total populations of nematodes over the two zones were summed and reported in Table 1. The results showed that, in the phytate medium, the bacterial strains did not modify the size of nematode populations; this is contrary to the Pi medium, where *Bradyrhizobium* sp. increased the numbers of nematodes significantly by 35%. The ratio of nematode populations counted in phytate to those counted in the Pi medium was always less than 1, indicating that Pi was a better source of P than phytate. However, this ratio was higher with *B. subtilis* 168 than with *Bradyrhizobium* sp., suggesting that the growth capacities of *B. subtilis* 168 on both sources of P were comparable, contrary to *Bradyrhizobium* developing better on Pi than on phytate.

**Table 1.** The total numbers of *Acrobeloides* sp. individuals according to the co-inoculated bacterial species, *B. subtilis* 168 or *Bradyrhizobium* sp., and the source of P supplied into the medium, phytate or Pi.

		Bacterial strains	
Parameters	Source of P	*B. subtilis* 168	*Bradyrhizobium* sp.
Numbers	Phytate	38.0 × 10^3^ ± 2.84 × 10^3bA^	39.7 × 10^3^ ± 1.42 × 10^3bB^
	Pi	45.9 × 10^3^ ± 2.28 × 10^3bA^	62.1 × 10^3^ ± 4.02 × 10^3aA^
Ratio	Phy/Pi	0.83 ± 0.07^a^	0.64 ± 0.03^b^

**Notes:** The ratio of individuals counted in the phytate and Pi medium for each bacterium is also given. Data are means (SE, *n* = 5). The letters indicate significant differences of means between the bacterial species (lowercase) or the source of P within a same bacterium treatment (uppercase) at *p* < 0.05.

We also calculated the nematode multiplication rates by dividing the total numbers of nematodes collected in both zones by the initial number inoculated in zone 1 (100 individuals) (Table 2). With phytate, the values did not significantly differ between bacterial strains; however, with Pi, nematodes feeding on *Bradyrhizobium* sp. multiplied faster (+35%) than on *B. subtilis* 168.

**Table 2.** Ratio of multiplication of *Acrobeloides* sp. fed on *B. subtilis* 168 or *Bradyrhizobium* sp. and grown with phytate or Pi as the sole source of P, calculated by dividing the total numbers of nematodes counted after three weeks by the initial number inoculated in zone 1 (100 individuals).

	Source of P	
Bacterial strains	Phytate	Pi
*B. subtilis* 168	379.9 ± 28.4^b^	459 ± 22.9^b^
*Bradyrhizobium* sp.	396.9 ± 14.2^b^	621 ± 40.2^a^
Bacterial effect		***

**Notes:** Data are means (±SE, *n* = 5). The letters indicate significant differences of means between the source of P within a same bacterium treatment at *p* < 0.05; stars (*p < 0.05; **p < 0.01; ***p < 0.001) indicate significant differences between bacterial strains (bacterial effect).

### Effect of nematodes and P sources on bacterial growth

Without nematodes, bacterial densities from both strains were much higher in zone 1 than in zone 2, irrespective of the P source and the bacterial strain (Table 3). However, within each P source, total bacterial populations differed according to the bacterial strain, with *B. subtilis* 168 developing more than *Bradyrhizobium* sp. (+46%) in the phytate medium (Table 4). The ratio of bacteria counted in the phytate medium to those counted in the Pi medium was very low for both bacterial species (Table 3), indicating that bacterial growth was much lower with phytate than with Pi, by a factor 10 for *Bradyrhizobium* and 5 for *B. subtilis 168*. When *Acrobeloides* sp. nematodes were inoculated in the medium, we observed significant bacterial growth in zone 2 (Table 3). Irrespective of the P source, *B. subtilis* 168 populations were greater than those of *Bradyrhizobium* sp. in zone 1 and not significantly different in zone 2 (Table 3). Total bacterial populations of *B. subtilis* 168 were always higher than those of *Bradyrhizobium* sp., both with phytate (+175%) and with Pi (+195%) (Table 4). Nematode grazing had different effects on bacterial populations, according to the P source and the bacterial strain, with (i) no effect on *B. subtilis* 168 and *Bradyrhizobium* sp. in phytate medium, and (ii) a very strong negative effect (by a factor of 5.5 for *B. subtilis* 168 and 15 for *Bradyrhizobium* sp. populations) in Pi medium (Table 4). Finally, the ratio of bacterial populations in the phytate medium to the Pi medium was not different between bacterial strains and was higher than one, indicating that the bacteria multiplied faster with phytate than with Pi with nematode grazing. Hence, the grazing effect on the phytate/Pi ratio was strongly positive for both bacterial strains (Table 4).

**Table 3.** Evolution of bacterial populations of *B. subtilis* 168 or *Bradyrhizobium* sp. throughout the zones 1 and 2 after inoculation at a single point located at the top of zone 1 either in absence (−N) or in presence (+N) of bacterial grazers.

		*B. subtilis* 168		*Bradyrhizobium* sp.	
P source	Bacterial grazers	Zone 1	Zone 2	Zone 1	Zone 2
Phytate	−N	9.08 × 10^6^ (1.90 × 10^6^)^a^	0.60 × 10^6^ (0.04 × 10^6^)^b**^	6.42 × 10^6^ (2.90 × 10^6^)^a^	0.20 × 10^6^ (0.09 × 10^6^)^b**^
Phytate	+N	5.50 × 10^6^ (0.85 × 10^6^)^a^	3.45 × 10^6^ (0.61 × 10^6^)^ab^	1.80 × 10^6^ (0.30 × 10^6^)^b^	1.52 × 10^6^ (0.20 × 10^6^)^b^
Pi	−N	27.2 × 10^6^ (12 × 10^6^)^a^	0.07 × 10^6^ (0.04 × 10^6^)^b**^	41.9 × 10^6^ (7 × 10^6^)^a**^	0.20 × 10^6^ (0.15 × 10^6^)^b**^
Pi	+N	3.40 × 10^6^ (0.38 × 10^6^)^a^	2.20 × 10^6^ (0.41 × 10^6^)^ab^	1.59 × 10^6^ (0.18 × 10^6^)^b^	1.59 × 10^6^ (0.29 × 10^6^)^b^

**Notes:** Data are means (SE, *n* = 5). The same lowercase letters indicate a lack of significance (*p* < 0.05) within the same zone of the Petri dish. Stars indicate a significant difference between the absence/presence of *Acrobeloides* sp. (*p < 0.05; **p < 0.01) for the same zone within a P source.

**Table 4.** Total bacterial cells according to the P source (phytate or Pi) and the absence (−N) or the presence (+N) of *Acrobeloides* sp.

			Bacterial strains	
Parameters	P source	Bacterial grazers	*B. subtilis* 168	*Bradyrhizobium* sp.
Numbers	Phytate	−N	9.67 × 10^6^ (1.81 × 10^6^)^a^	6.62 × 10^6^ (2.13 × 10^6^)^b^
	Phytate	+N	8.92 × 10^6^ (1.01 × 10^6^)^a^	3.28 × 10^6^ (0.43 × 10^6^)^b^
	Grazing effect	NS	NS	
	Pi	−N	28.1 × 10^6^ (13.7 × 10^6^)^a^	42.2 × 10^6^ (8.87 × 10^6^)^a^
	Pi	+N	5.44 × 10^6^ (0.51 × 10^6^)^a^	2.84 × 10^6^ (0.46 × 10^6^)^b^
	Grazing effect	*	***	
Ratio	Phytate/Pi	−N	0.20 ± 0.06^b^	0.11 ± 0.04^b^
	Phytate/Pi	+N	1.63 ± 0.12^a^	1.35 ± 0.04^a^
	Grazing effect	***	**	

**Notes:** The ratio of bacteria counted in the phytate and Pi medium with or without nematodes is also given. Data are means (SE, *n* = 5). The letters indicate significant differences of means between the bacterial species within each condition (*p* < 0.05); stars (*p < 0.05; **p < 0.01; ***p < 0.001) indicate significant differences between presence and absence of nematodes (grazing effect).

### Pi release from phytate according to the presence/absence of bacterial grazers

**Figure 3:**
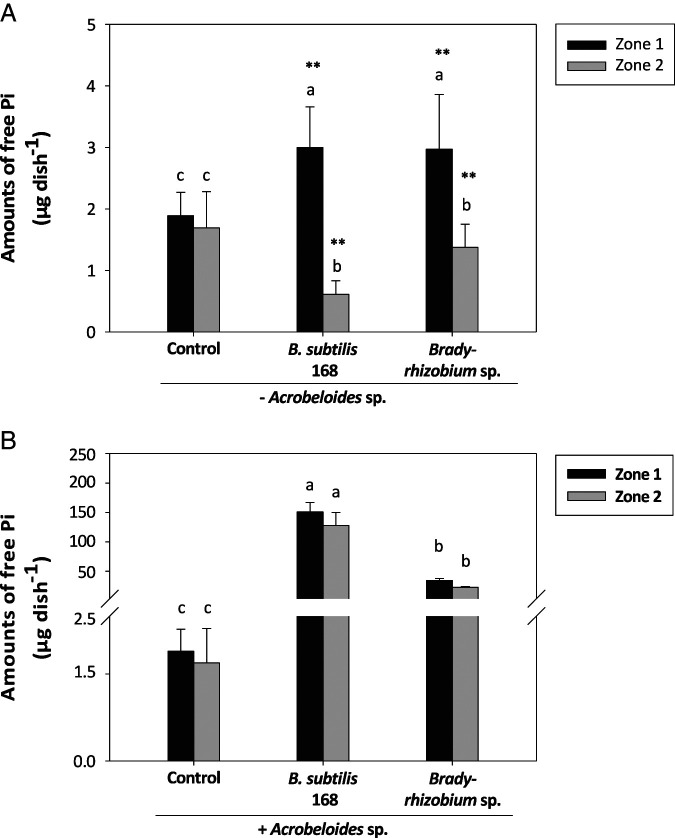
Effect of grazing activities by *Acrobeloides* sp. nematodes on phytate mineralization catalyzed either by *B. subtilis* 168 or *Bradyrhizobium sp*. Shown are the free Pi amounts extracted from zones 1 or 2 after three weeks of incubation with phytate as the sole source of P in absence (A) or in presence (B) of nematodes. Bacteria (A) or nematodes (B) were inoculated at a single point located at the top of zone 1 with nematodes previously fed on each bacterial strain. Control treatment (Ctrl) consisted of water deposit instead of bacteria or nematodes. Data are means (±SE, *n* = 5). The same lowercase letters indicate a lack of significance (*p* < 0.05) within the same zone of the Petri dish. Stars above a bar indicate a significant difference between the absence/presence of *Acrobeloides* sp. (*p < 0.05; **p < 0.01) within each zone and bacterial strain.

When no organism was added into the Petri dish containing phytate as the sole source of P (control treatment in Fig. 3A, B), the amounts of free Pi extracted from each zone were low and similar, with values <2 µg per zone, indicating that the Na-phytate and the agarose used in this experiment contained only trace portions of free Pi. Without *Acrobeloides* sp., the addition of bacteria increased the amounts of free Pi extracted from zone 1, but only slightly: the values reached up to 3 µg for both bacterial strains (Fig. 3A). However, in zone 2, the amounts of free Pi significantly decreased compared to control conditions for both bacterial strains (Fig. 3A). When *Acrobeloides* sp. was co-inoculated with *B. subtilis* 168 or *Bradyrhizobium* sp., the free Pi content strongly increased (Fig. 3B). The interaction between *Acrobeloides* sp. and *B. subtilis* 168 resulted in values up to 150 µg in zone 1 and 127 µg in zone 2, respectively; values that were strongly different compared to *Acrobeloides* sp. fed on *Bradyrhizobium* sp., which had 34 µg in zone 1 and 22 µg in zone 2 (Fig. 3B). To evaluate the effects of grazing activities on Pi release by each bacterial strain, we divided the total amount of Pi released per dish by the total number of bacterial cells (Table 4). Without nematodes, the two bacterial strains released not significantly different amounts of Pi per million cells. However, when *Acrobeloides* sp. was present, *B. subtilis* 168 released more Pi than *Bradyrhizobium* sp. (+55%). Hence, for both bacterial strains, the free Pi amounts measured after grazing by nematodes were significantly increased compared to the values measured without nematodes. However, this positive effect on Pi release was much stronger when *Acrobeloides* sp. was fed on *B. subtilis* 168 than on *Bradyrhizobium* sp., with factors of 30 and 5, respectively (Table 5).

**Table 5.** Total amounts of free Pi mineralized by each bacterial strain and expressed in µg of free Pi per million of bacterial cells grown in absence (−N) or in presence (+N) of nematodes.

	Bacterial strains	
Bacterial grazers	*B. subtilis* 168	*Bradyrhizobium* sp.
−N	0.95 ± 0.21^a^	3.71 ± 1.73^a^
+N	31.26 ± 3.67^a^	18.96 ± 0.86^b^
Grazing effect	***	***

**Notes:** Data are means (±SE, *n* = 5). The letters indicate significant differences of means between the bacterial species within each condition (*p* < 0.05); stars (*p < 0.05; **p < 0.01; ***p < 0.001) indicate significant differences between the presence and absence of nematodes (grazing effect).

## Discussion

Our study showed that *Acrobeloides* sp. nematodes fed on *B. subtilis* 168 or on *Bradyrhizobium* sp. were able to migrate from the inoculation point to colonize the second compartment of the agarose medium after three weeks of monoxenic culture. However, the size and the multiplication rates of the total populations of nematodes depended simultaneously on the bacterial strain and the source of P, as they were the highest when the source of food was *Bradhyrhizobium* sp. supplied with Pi. This indicates that, in non-limiting conditions, *Bradhyrhizobium* sp. was a better source of food for *Acrobeloides* sp. than *B. subtilis* 168. Our results are in line with previous experimental data suggesting that some species of bacterivores can distinguish between bacteria and preferentially consume some of them (Blanc et al., 2006); and those found by Liu et al. (2017) who reported better preferences of *Acrobeloides* sp. for feeding on Gram – than Gram+bacteria. This feeding behavior of *Acrobeloides* sp. could be related to its buccal morphology as it will determine its ability to capture and ingest bacterial cells of different sizes and mobility (Bongers and Ferris, 1999). The *Acrobeloides* sp. nematodes used in our study belong to the *Cephalobidae* family and have a funnel-shaped buccal morphology (Ferris et al., 1996) that is more adapted to ingest smaller prey than larger. In our study, *B. subtilis* 168 had a larger size and a thicker cell wall than *Bradyrhizobium* sp. since they belong to Gram+ and Gram− classes, respectively. In fact, the large-sized and thicker cell-walled bacteria are considered to be low-quality food and hard to pass through the narrow buccal cavity (Avery and You, 2012; Salinas et al., 2007), which might determine the ability of *Acrobeloides* sp. to find and selectively ingest bacterial species such as *Bradyrhizobium* sp. The low bacterial cell density found for *Bradyrhizobium* sp. well supports the hypothesis that the thinner cell walls of *Bradyrhizobium* sp. are easier to ingest and digest with respect to *B. subtilis* 168. Besides these factors, we cannot exclude that the feeding behavior of *Acrobeloides* sp. could also be influenced by some compounds in the medium, such as ions, amino-acids, vitamins, CO_2_, and/or pH gradient, as proposed by Rasman et al. (2012), influencing, in turn, its growth and reproduction rates (Sohlenius, 1973).

When phytate was supplied as the sole source of P, the populations of nematodes did not differ between both bacterial strains in each zone and, in zone 2, were roughly half of those in zone 1. At first glance, this suggests that the final population density was limited by bacterial growth. This hypothesis is supported by the total number of bacterial cells counted without nematodes, which was much lower with phytate than with Pi, for both bacterial strains. This lower bacterial growth might be due to the time required to induce the expression of phytase enzymes, as shown for *B. subtilis* (Powar and Jagannathan, 1982). However, relative to Pi, the negative effect of phytate on bacterial growth was stronger on *Bradyrhizobium* sp. than on *B. subtilis* 168. These differences between the two bacterial species could be due to a lesser efficiency of *Bradyrhizobium* sp. in using phytate as a sole source of P than *B. subtilis* 168. Whereas it is well known that *B. subtilis* 168 is able to express and secrete a BPP phytase into the external media (Chen et al., 2015; Jorquera et al., 2013; Lim et al., 2007), the type of phytase produced by *Bradyrhizobium* sp. has not been described yet. Our results demonstrated that this bacterial strain is able to use phytate as the sole source of P. This ability could result from the production of a phytase enzyme that might be homologous to the protein with an esterase-like activity of the phytase family identified in the genome *B. japonicum* (strain USDA6^T^) (Kaneko et al., 2011). Compared with *B. subtilis* 168, this phytase could not be released into the external medium but could have an intracellular or periplasmic localization (Lim et al., 2007), together with several different biological properties in comparison with the *B. subtilis* BPP phytase. Such enzyme differences would explain the lower ability of *Bradyrhizobium* sp. to use phytate compared to Pi than *B. subtilis* 168.

A way to estimate the efficiency of phytate mineralization by bacteria for plant nutrition is to measure the net release of free Pi into the medium. Without nematodes, both bacterial species induced opposite trends on the free Pi according to the zone. In zone 1, free Pi increased slightly whereas, in zone 2, it decreased compared to control conditions (phytate medium, not inoculated). The small Pi increase observed in zone 1, which was highly colonised by bacterial cells, could reflect either a low phytase activity or a strong immobilization of free Pi into the bacterial biomass whose populations are not N- or C-limited. Conversely, the sizes of bacterial populations in zone 2 were much smaller than in zone 1, probably because the bacterial growth was delayed compared to zone 1. If it started during the last days of the experiment, the decrease of free Pi could correspond to Pi immobilization into bacterial cells in order to induce enzyme production, resulting in a Pi decrease in the medium. Also, when we expressed the total amounts of free Pi released per millions of bacteria, we found similarly low values for both bacterial strains, suggesting that the two bacterial species have the same efficiency to take up the free Pi from the medium. In contrast, when bacteria and nematodes were co-inoculated, the amounts of free Pi were similar between both zones and much greater for *B. subtilis* 168 than for *Bradyrhizobium* sp. Thus, our results can explain the beneficial effects of nematodes on plant P acquisition from phytate mineralized by *B. subtilis* reported by Irshad et al. (2012), since the migration of nematodes will allow the proliferation of bacteria throughout the whole medium, thus increasing the probability of phytate hydrolysis by bacterial phytases. In addition to the increase of the absolute amount of free Pi released into the medium, the nematode activity also increased the free Pi amounts expressed per millions of bacteria, and this effect was higher for *B. subtilis* 168 than for *Bradyrhizobium* sp., indicating that the presence of bacterial grazers definitely induced more Pi release with *B. subtilis* 168 than with *Bradyrhizobium* sp. This enhancement of Pi release per cell unit following nematode inoculation could be due to the production of phytase by the nematodes. Although genomic data available for nematodes (Howe et al., 2017) or *Acrobeloides nanus *(Schiffer et al., 2018) did not retrieve any hit for phytase, we found a protein in the genome of *Caenorhabdtidis elegans* displaying homology with the phytase from *Aspergillus niger* (PhyA, NCBI reference sequence XP_001401713.2). This protein (PHO-13, WormBase ID: CE35850) might hydrolyze phytate and raised therefore the question whether or not the nematodes are able to hydrolyze phytate by themselves. Alternatively, the specific effect of grazing of *Acrobeloides* sp. on phytate mineralization by *B. subtilis* 168 might result from two possible effects. Firstly, it could be a greater or faster turnover rate of locked P in *B. subtilis* 168 than in *Bradyrhizobium* sp. Secondly, the presence of *Acrobeloides* sp. could enhance the release of the BPP phytase by *B. subtilis* 168. This hypothesis is supported by recent experiments showing that the arbuscular mycorrhizal fungus *Rhizophagus irregularis* increased the expression of the phosphatase gene and the release of phosphatase activity by the bacterium *Rahnella aquatilis* and, thus, the mineralization of phytate (Zhang et al., 2018). In the future, our experimental system could be used to assess the effect of other nematode species, whose genome resources are available, on phytate mineralization by a bacterium such as *B. subtilis* or other bacterial species with available genomic data. The experimental data could then be followed up by transcriptomic studies performed on worms and bacteria simultaneously using dual RNAseq (Westermann et al., 2012). This new tool might enable us to elucidate the molecular mechanisms behind the changes we observed in phytate mineralization when the worm is added into the medium.

## Conclusions

Our work brought new insights into the specific mechanisms driving phytate mineralization through the predation activities of bacterial grazers. Firstly, bacterial grazing was accompanied by the migration of nematodes, allowing the proliferation of bacteria throughout the whole medium and increasing the probability of phytate hydrolysis by bacterial phytases. Secondly, despite the grazing preference of *Acrobeloides* sp. for *Bradhyrhizobium* sp. (Gram−) compared to *B. subtilis* 168 (Gram+), this interaction resulted in a much stronger increase of Pi release from phytate by *B. subtilis* 168 than by *Bradhyrizobium* sp. Thus, our results can explain the beneficial grazing effects of nematodes on plant P acquisition from phytate mineralized by *B. subtilis* 168 reported previously. Thirdly, when calculated per million of bacteria, the amounts of free Pi released in the presence of *Acrobeloides* sp. were significantly enhanced compared to those measured in the absence of nematodes, especially for *B. subtilis* 168. *Acrobeloides* sp. could, therefore, specifically increase the expression of the BPP gene, and this would explain the strong positive effect of nematode grazing on phytate mineralization by *B. subtilis*. Though this hypothesis needs to be checked, our results showed that the bacterial grazers predation activities drove the specific mechanisms within its trophic relationship with *B. subtilis* 168. Hence, using these grazing activities in a better way could represent a suitable alternative for recycling Po sources in soils that are currently poorly available to plants.
